# Resilience and Stress Among Health Care Workers Participating in the StressPal Frontline Program: Quasi-Experimental Pretest-Posttest Study

**DOI:** 10.2196/85388

**Published:** 2026-07-08

**Authors:** Debora Goetz Goldberg, James Monroe, Pennie Sempell, Jehanzeb Cheema, Sravya Vunnam, Panagiota Kitsantas

**Affiliations:** 1 Department of Health Administration, Policy, and Informatics, College of Public Health, George Mason University, Peterson Health Sciences Hall, 4400 University Drive MS IJ3, Fairfax, VA, 22030-4422, United States, 1 2024958441; 2 Center for Evidence-Based Behavioral Health, Department of Psychology, George Mason University, 4400 University Drive, Fairfax, VA, 22030, United States, 1 703-993-1384; 3 StressPal, Inc., San Rafael, CA, United States; 4 Department of Information Systems and Operations Management, Costello College of Business, George Mason University, Fairfax, VA, United States

**Keywords:** acceptance and commitment therapy, ACT, digital interventions, mental health, mindfulness-based stress reduction, MBSR, mindfulness-based cognitive therapy, MBCT, mobile health app, occupational stress, psychological flexibility, professional burnout

## Abstract

**Background:**

Health care workers continue to experience heightened levels of distress and burnout, which contribute to higher levels of job dissatisfaction, turnover intentions, presenteeism, and staffing shortages.

**Objective:**

The aim of this study was to examine how participation in the StressPal Frontline: Essential Resilience Self-Care and Burnout Prevention program influenced health care workers’ stress and resilience. The study also sought to identify specific measures of perceived stress and resilience that were most affected by participation in the program and to explore whether pre-and-post differences varied based on participant characteristics. The StressPal Frontline program is a digital resilience intervention specifically developed for health care workers to enhance psychological flexibility and stress resilience. The self-paced training program, designed for approximately a 6-week period, consists of brief modules, follow-up resources, and a peer engagement community.

**Methods:**

A pretest-posttest quasi-experimental design was used to assess the effectiveness of the StressPal Frontline program in reducing stress and building resilience among 76 health care workers who voluntarily joined and completed the program. Outcome measures included the Perceived Stress Scale and the Brief Resilience Scale to assess participants’ perceptions of stressful situations and their ability to bounce back from stress. Descriptive statistics, correlation analysis, paired-samples 2-tailed *t* test, and multiple regression analysis were conducted. The paired-samples *t* test was calculated at the scale level and item level to evaluate the statistical significance of pretest and posttest mean differences, and the Cohen *d* statistic was used as a measure of effect size. Statistical analysis for this study was conducted in Excel (Microsoft), SPSS (IBM Corp), and Jamovi (jamovi project).

**Results:**

The results indicated a 1.53-point reduction in the Perceived Stress Scale score after participating in the StressPal Frontline program, suggesting a statistically significant decline in average perceived stress due to participation in the program (*P*=.004). The corresponding value of Cohen *d* was 0.34, suggesting a small-to-medium effect of the intervention, StressPal Frontline program, in reducing perceived stress. For the Brief Resilience Scale, pre-and-post difference was not significant at the scale level (*P*=.07); however, item-level analysis found significant increases in participants’ perception of their ability to bounce back quickly after hard times and handle difficult situations. No significant differences were found in outcome measures based on age, race, ethnicity, professional role, or practice setting.

**Conclusions:**

The StressPal Frontline program was associated with positive outcomes in reducing perceived stress. Our study also found no statistical differences in outcomes among participants of different age groups, races, ethnicities, occupations, genders, and practice settings. This is an important finding, as it indicates that the StressPal Frontline program may provide positive benefits for reducing stress across professions, settings, and individual characteristics. This program, along with other resources, could be implemented by health care organizations to support workers’ professional development, behavioral health, and well-being.

## Introduction

### Occupational Distress and Resilience-Building Approaches

Concerns regarding high levels of occupational distress among health care professionals predated the pandemic; however, workforce strain and intent-to-leave indicators remain higher than prepandemic baselines, with workforce shortages expected to continue through 2037 [1,2]. Previous research has demonstrated a strong association between occupational distress and burnout, which has been linked to increased absenteeism and presenteeism, reduced productivity and engagement, and issues with the quality of patient care [3-5].

Health care delivery is inherently complex, unpredictable, and challenging. Frontline health care providers and staff are faced with multifaceted responsibilities, complex job tasks, and competing demands that can lead to stress, fatigue, and burnout. Other external and systemic organizational-level factors and system inefficiencies drive higher rates of distress and burnout. Workplace issues, such as harassment, violence, and individual vulnerabilities, create further complexities, impacting individual well-being, communication, work performance, safety, and job satisfaction [6-10].

The high prevalence of stress and stress-triggered responses (cognitive, behavioral, and physical) among health care workers remains a critical problem [11,12]. Research over the past several decades has established the detrimental impact of chronic stress. At fundamental levels of stress physiology, sustained activation of the stress response, notably through cortisol release, can lead to adverse health outcomes, particularly when psychosocial resilience is insufficient. The COVID-19 pandemic compounded challenges to health care workers’ resilience, manifesting in a multitude of stress-induced conditions, including anxiety, depression, emotional dysregulation, exhaustion, and professional dissatisfaction [12]. These conditions continue to negatively impact the well-being, quality of life, burnout, retention, and job performance among health care workers [8,11].

There is extensive evidence that supports brief interventional training approaches to build psychological flexibility and resilience [13-16]. The benefits of mindfulness, psychological flexibility, and resilience include enhanced performance, such as creative problem-solving and teamwork, as well as interpersonal behaviors such as improved cognitive functioning, energy, focus, compassion, and self-compassion, among a wide range of other mental and physical benefits [17,18]. There is also a large body of research on the benefits of and the methodology for intentional learning to (1) cultivate the brain’s capacity for psychological flexibility and stress resilience, (2) defuse the stress response, and (3) commit to choosing effective stress-triggered responses in the heat of the moment [19-22].

### Mindfulness-Based Interventions for Stress and Burnout

During the period 1982 to 2017, research on mindfulness-based interventions for stress resilience was conducted primarily among non–health care workers. These efforts included considerable attention to mindfulness-based stress reduction (MBSR) interventions [23-34]. There continues to be a high number of studies focused on MBSR interventions, including their expansion to health care workers [35]. Time requirements for completing the full MBSR program, which is typically 2.5 hours per week for 8 weeks, have raised concerns regarding attrition rates among health care study participants. These concerns have led to the development of shortened MBSR programs and alternative mindfulness-based interventions tailored for health care settings [36,37].

### Acceptance-Based Mindfulness Models

Substantial empirical evidence supports the integrative efficacy of acceptance and commitment therapy (ACT) [38-46], particularly its core processes of acceptance, psychological flexibility, and engagement in valued actions. ACT interventions aimed at facilitating psychological flexibility are also considered deliverable in time-efficient ways. Acceptance-based training models, mindfulness-based cognitive therapy (MBCT) [36,42,43], and mindful self-compassion [46,47] have shown favorable outcomes for stress resilience, work-life balance, well-being, and the reduction of symptoms of burnout among health care workers. Such models elucidate foundational mindfulness processes of awareness with nonreactive, nonjudgmental, present-moment acceptance of thoughts, feelings, behaviors, and physical responses as mediators of change. Studies have further demonstrated that such mindfulness processes are not only theoretically integral to but are also therapeutically strengthened by the core processes of psychological flexibility and valued actions, both defining constructs of ACT [42]. Prior studies of mindfulness-based ACT processes in the health care workforce found that specific interventions resulted in significant improvements in psychological flexibility, reductions in perceived stress and burnout, and increased resilience and overall well-being [48,49].

Contemporary versions of cognitive behavioral therapy (CBT) and mindfulness processes [50], such as ACT and MBCT, have highlighted a shift toward process-based therapy. These approaches focus on altering one’s relationship with internal experiences and emphasize contextual and functional change processes [51], which promote a broader integration of therapeutic processes targeted to mediate psychological flexibility.

### Development of the StressPal Frontline Program

The StressPal Frontline: Essential Resilience Self-Care and Burnout Prevention (hereafter referred to as StressPal Frontline) is a comprehensive digital resilience training program and peer engagement community specifically developed for health care workers to enhance psychological flexibility and stress resilience. The program was developed by a multidisciplinary team at StressPal, which was led by a clinical psychologist with a specialty in stress-related and anxiety disorders. The development team also included specialists in health education and conflict resolution, who worked closely with professionals in medicine, nursing, distance education, information technology, and media production. The program, designed for approximately a 6-week period, is self-paced and consists of brief modules and follow-up resources provided on a digital learning platform. StressPal Frontline integrates process-based therapeutic models from ACT, CBT, and MBCT. The program trains participants in adaptive responses to stress and internal experiences by cultivating nonreactivity, nonjudgment, and value-driven behavior.

The conceptual and content development of the StressPal Frontline program was informed by a comprehensive review of mindfulness-based ACT and CBT-based interventions, with a primary focus on training stress resilience for health care workers. Methods for training participants in mindfulness strategies and skills were enhanced through concise incremental training steps and modeling of integrated mindfulness processes, such as acting with awareness, observing, describing, acceptance (ie, willingness to experience distress), cognitive defusion (eg, creating distance from unhelpful thoughts), valued actions (eg, aligning actions with personal values), and self-awareness and attention. The program draws on validated frameworks such as the Five Facet Mindfulness Questionnaire [23] and MBCT [52], and includes techniques such as behavioral activation, Socratic questioning, and applications of metaphors (Table 1).

**Table 1.** Core interventional processes in the StressPal Frontline: Essential Resilience Self-Care and Burnout Prevention program.

Methodology	ACT^a^ model	CBT^b^ or MBCT^c^ process	Key concepts included in the StressPal Frontline program
Mindfulness	Self-as-context; self-as-observer (ACT)	Decentering, observing, and describing (MBCT); acting with awareness (FFMQ^d^)	Nonreactive, present-focused attention; stepping back from thoughts
Acceptance	Willingness to experience discomfort	Behavioral exposure to reduce avoidance	Nonjudgmental openness to thoughts and feelings
Cognitive defusion	Metaphors	Socratic questioning	Observing thoughts without attachment or judgment
Valued directions and actions	Committed action in service of personal values	Behavioral activation of meaningful activities	Values-guided actions; prosocial behaviors
Awareness and attention	Self-as-observer (nonjudging perspective)	Decentering from automatic thoughts; reframing	Observing internal experiences without overidentifying

a ACT: acceptance and commitment therapy.

b CBT: cognitive behavioral therapy.

c MBCT: mindfulness-based cognitive therapy.

d FFMQ: Five Facet Mindfulness Questionnaire.

The StressPal Frontline program includes a wide breadth of approaches to stress resilience training to meet the needs of individuals from various health care occupations, cultural backgrounds, and practice settings. Strategies focus on problem-solving, conflict resolution, and mindfulness-based advancement of supportive communication practices, such as openness, honesty, and compassion [53-56]. Engagement, retention, and program adherence are enhanced with the use of action-based learning methods, personalized life-practice reporting, animated storytelling, game-like features, and automated reminders. Story themes unfold, depicting a range of roles, ages, genders, and ethnicities typical of health care settings. Together, these present a learning experience in modeling optional processes for psychological flexibility and mindful awareness of stress-triggered responses. The program was developed and beta-tested during the COVID-19 pandemic and has been deployed in various health care settings, including hospitals and health centers, and evaluated with self-report and open-text items.

### Study Objectives

The purpose of this study was to (1) examine whether participation in the StressPal Frontline program influenced perceived stress and resilience among health care professionals; (2) identify specific measures of perceived stress and resilience that were most affected by the StressPal Frontline program; and (3) explore whether pre-and-post differences in perceived stress and resilience varied based on participant characteristics such as professional role, practice setting, gender, race, ethnicity, and age.

## Methods

### Research Design

A pretest-posttest quasi-experimental design was used to assess the effectiveness of the StressPal Frontline program in reducing stress and building resilience among health care workers who completed the program. ChatGPT (GPT-5.5 Instant; OpenAI) was used to identify some of the peer-reviewed literature cited in this paper. Our research team retrieved each paper suggested by ChatGPT and reviewed its content. Other peer-reviewed literature was identified through searches conducted using PubMed, APA PsycInfo (EBSCO), CINAHL Complete, and other library databases.

### Ethical Considerations

Ethics approval for this study was granted by the George Mason University institutional review board in September 2022 (institutional review board number 1925894).

### Sample

As part of the enrollment process, training participants were informed of data security protocols and confidentiality procedures. Participants were recruited through announcements issued by health care employers and affiliated professional associations. The announcements provided a link to a web page and an online application. On a first-come, first-served basis, applicants were included in the study if they were aged 18 years or older, worked in health care (clinical or nonclinical roles), and were residents of the United States. Applicants who met the inclusion criteria received an invitation email with a unique URL and access code, which they could use to sign up for the web-based StressPal Frontline program.

### Data Analysis

The Perceived Stress Scale (PSS) [57] and the Brief Resilience Scale (BRS) [58] were used to evaluate the effectiveness of the StressPal Frontline program. The PSS is the most widely used psychological instrument for measuring the perception of stress and includes 10 items on current levels of experienced stress. PSS scores are obtained by reversing responses to the 4 positively stated items and then summing across all scale items. An example item included “In the last month, how often have you been upset because of something that happened unexpectedly?” Higher PSS scores are indicative of higher levels of perceived stress. The BRS assesses an individual’s ability to recover from adversity. It includes 6 self-reported items rated on a 5-point agreement scale ranging from 1 (strongly disagree) to 5 (strongly agree). A sample item included “I tend to bounce back quickly after hard times.” These scales were administered to all participants before and after the StressPal Frontline intervention.

Statistical analysis for this study was conducted using Excel (Microsoft), SPSS (IBM Corp.), and Jamovi (jamovi project). The analysis was performed by a team member who was not involved in implementing the intervention and knew participants only by their study identifier numbers.

The paired-samples *t* test at scale-level and item-level measures was used to evaluate the statistical significance of pretest and posttest mean differences. In order to evaluate the practical importance of statistically significant results, Cohen *d* was calculated and interpreted as a measure of effect size [59]. The relationship between changes in scale scores and 5 individual participant characteristics (gender, race, ethnicity, role, and setting) was also examined, both individually (bivariate analysis: independent-samples *t* test, Pearson *r*, and Mann-Whitney *U* test) and collectively (multiple regression analysis). The analyses outlined in this section were performed separately for PSS and BRS. For each analytical method, the underlying model assumptions were evaluated, and a 5% level of significance was used for the evaluation of hypotheses.

A paired-samples *t* test was performed comparing the average pretest and posttest PSS scores, with the analysis repeated for BRS. The paired-samples *t* test procedure was repeated for each of the 10 individual PSS items to evaluate the statistical significance of individual items. The main aim of this analysis was to determine if a subset of items was behaving abnormally relative to the overall scale.

The movement of participants across resilience categories (low, normal, and high) [60] was also examined to determine if the intervention had a significant impact on stress by examining whether a significant number of participants moved down from one category to a lower category (ie, did they move from high to moderate or from moderate to low).

A bivariate analysis of PSS and BRS pre-and-post difference scores was conducted to evaluate differences based on demographic variables. Then, a multiple regression model with the PSS difference score as the outcome in one model and the BRS difference score as the outcome in another model was conducted to evaluate whether the changes observed in the pretest and posttest differed significantly while holding all else constant.

## Results

There were 148 health care professionals who started the StressPal Frontline program between February 2024 and February 2025 and completed pretraining assessments; 76 completed the program and were included in the analysis. A majority of participants self-identified as female participants (n=66, 86.8%), not of Hispanic or Latino origin (n=70, 92.1%), and White (n=58, 76.3%). Participant age ranged from 27 to 72 (mean 45.62, SD 10.98) years. The top 3 profession or role categories were as follows: (1) nurse (n=37, 48.7%), (2) mental health or allied professional (n=12, 15.8%), and (3) advanced practice provider or professional (n=8, 10.5%). The top 3 settings included urban or suburban hospitals (n=20, 26.3%), other health care settings such as physician offices and free clinics (n=19, 25%), and community-based organizations or public health settings (n=14, 18.4%). There was no significant difference between those who completed the program and those who did not based on professional role or practice setting.

Descriptive statistics for the PSS scale and items are presented in Table 2. These figures show that, on average, the posttest score for each individual item in the PSS was lower than its pretest counterpart. Descriptive statistics for the BRS scale and items are presented in Table 3. These figures show that, on average, the posttest score for each individual item was either the same or higher than its pretest counterpart.

**Table 2.** Summary statistics and paired-samples *t* test results for the Perceived Stress Scale (N=76).^a^

Item description	Pretest, mean (SD)	Posttest, mean (SD)	Δ*M*^b^	95% CI^c^	Cohen *d*^d^
Overall scale^e^	19.74 (6.22)	18.21 (6.77)	−1.53^f^	−2.56 to −0.49	−0.34
In the last month, how often have you:					
1. been upset because of something that happened unexpectedly?	2.34 (0.78)	2.20 (0.75)	−0.14	−0.34 to 0.06	−0.17
2. felt that you were unable to control the important things in your life?	2.14 (0.98)	2.04 (0.97)	−0.11	−0.29 to 0.08	−0.13
3. felt nervous and stressed?	2.76 (0.89)	2.45 (0.94)	−0.32^f^	−0.52 to −0.11	−0.35
4. felt confident about your ability to handle your personal problems?^g^	1.43 (0.87)	1.34 (0.86)	−0.09	−0.29 to 0.11	−0.10
5. felt that things were going your way?^g^	1.72 (0.93)	1.66 (0.93)	−0.07	−0.27 to 0.14	−0.07
6. found that you could not cope with all the things that you had to do?	1.88 (1.03)	1.76 (1.03)	−0.12	−0.34 to 0.11	−0.12
7. been able to control irritations in your life?^g^	1.62 (0.83)	1.61 (0.78)	−0.01	−0.22 to 0.19	−0.01
8. felt that you were on top of things?^g^	1.74 (0.85)	1.55 (0.86)	−0.18^h^	−0.36 to −0.01	−0.24
9. been angered because of things that happened that were outside of your control?	2.20 (0.85)	2.00 (0.92)	−0.20	−0.42 to 0.02	−0.21
10. felt difficulties were piling up so high that you could not overcome them?	1.89 (1.11)	1.61 (1.01)	−0.29^f^	−0.49 to −0.09	−0.33

a Response choices: 0 (never), 1 (almost never), 2 (sometimes), 3 (fairly often), and 4 (very often). Cronbach α: pretest 0.87; posttest 0.91.

b Δ*M *= *M*_posttest_* – M*_pretest_.

c The 95% CI is for the mean difference.

d Cohen (1992) guidelines for *d*: 0.2 (small); 0.5 (medium); 0.8 (large).

e The values are scale statistics.

f *P*<.01.

g Items 4, 5, 7, and 8 are reverse-coded; thus, higher item and scale values are indicative of higher stress.

h *P*<.05.

**Table 3.** Summary statistics and paired-samples *t* test results for the Brief Resilience Scale (N=76).^a^

Item description^b^	Pretest, mean (SD)	Posttest, mean (SD)	Δ*M*^c^	95% CI^d^	Cohen *d*^e^
Overall scale^f^	3.24 (0.75)	3.36 (0.73)	0.12	−0.01 to 0.26	0.21
1. I tend to bounce back quickly after hard times	3.39 (0.88)	3.58 (0.88)	0.18^g^	~0 to 0.37	0.23
2. I have a hard time making it through stressful events.	3.36 (0.93)	3.39 (0.98)	0.04	−0.16 to 0.24	0.05
3. It does not take me long to recover from a stressful event.	3.12 (0.91)	3.29 (0.88)	0.17	−0.06 to 0.40	0.17
4. It is hard for me to snap back when something bad happens.	3.24 (1.02)	3.24 (0.89)	0	−0.22 to 0.22	0
5. I usually come through difficult times with little trouble.	2.97 (0.88)	3.24 (0.88)	0.26^g^	0.05 to 0.47	0.29
6. I tend to take a long time to get over setbacks in my life.	3.36 (1.00)	3.43 (0.97)	0.08	−0.15 to 0.31	0.08

a Response choices: 1 (strongly disagree), 2 (disagree), 3 (neutral), 4 (agree), and 5 (strongly agree). Cronbach α: pretest 0.88; posttest 0.89.

b Items 2, 4, and 6 are reverse-coded; thus, higher item and scale values are indicative of higher resilience.

c Δ*M* = *M*_posttest_ – *M*_pretest_.

d The 95% CI is for the mean difference.

e Cohen (1992) guidelines for *d*: 0.2 (small); 0.5 (medium); 0.8 (large).

f The values are scale statistics.

g *P*<.05.

The *t* test results (reported in Table 2) indicated a 1.53-point reduction in PSS score after the StressPal Frontline program, suggesting a statistically significant decline in average perceived stress due to participation in the training (*P*=.004). The corresponding value of Cohen *d* was 0.34, suggesting a moderate (small-to-medium) effect of the intervention, the StressPal Frontline program, in reducing perceived stress. For BRS, pre-and-post difference was not significant at the scale level (*P*=.07).

Results of the paired-samples *t* test procedure for each of the 10 individual PSS items suggest that the significant intervention effect that we observed at the scale level can be traced to items 3, 8, and 10. For each of these items, on average, there was a statistically significant negative difference between posttest and pretest scores, suggesting a lower level of perceived stress at the conclusion of the StressPal Frontline program. The magnitude of the effect size (Cohen *d*) for these 3 items ranged from 0.24 to 0.35, which can be interpreted as small to medium. Thus, items 3, 8, and 10 were the main contributors to the decline in PSS scores as a direct result of the StressPal Frontline program. In other words, at the conclusion of the training, an average participant reported (1) a lower frequency of feeling nervous and stressed, (2) a higher frequency of feeling that they were on top of things, and (3) a lower frequency of feeling that difficulties were piling so high that they could not overcome them. We emphasize that while statistically significant, the corresponding effect sizes were moderate.

For the PSS scale, the movement of participants across stress categories (low, moderate, and high) was also examined [60]. Out of 10 respondents who reported high stress at pretest, 6 moved to the moderate category at posttest; out of 53 respondents in the moderate stress category at pretest, 11 moved to the low group while 3 moved to the high group; and out of the 13 participants in the low stress category at pretest, 2 moved to the moderate group. Thus, among the 76 participants, 22% (n=17) reported a reduction in perceived stress, 7% (n=5) an increase, while the remaining 71% (n=54) reported no change. The Wilcoxon signed-rank test indicated that this movement of participants across categories was statistically significant (*P*=.02). These results are in line with those from the pretest-posttest and suggest a moderate reduction in perceived stress among the participants.

For the BRS scale, although the scale-level pre-and-post difference did not reach a level of statistical significance, 2 of the 6 items (items 1 and 5) showed a statistically significant difference (Table 3), including participants’ ability to “bounce back quickly after hard times” and “come through difficult times with little trouble.” Three items trended toward a higher level of resilience, and 1 item showed no change—although none of these 4 differences were statistically significant (all *P*>.05). This sort of contradictory result can be obtained when positive and negative item effects cancel out each other and return an overall insignificant result at the scale level. Observation of this result highlights the importance of conducting item-level analysis. For items 1 and 5, on average, there was a statistically significant positive difference between posttest and pretest scores, suggesting a higher level of resilience at the conclusion of the StressPal Frontline program. The magnitude of the effect size (Cohen *d*) for items 1 and 5 was 0.23 and 0.29, respectively, which can be interpreted as small to medium. In other words, at the conclusion of the program, an average participant reported a higher level of agreement with the statements: (1) “I tend to bounce back quickly after hard times,” and (2) “I usually come through difficult times with little trouble.” While the pre-and-post differences on these items were statistically significant, we note that the corresponding effect sizes were moderate.

In our examination of the movement of participants across resilience categories (low, normal, and high) [60] on the BRS scale, out of 8 respondents who reported high resilience at pretest, 4 moved to the normal category at posttest; out of 42 respondents in the normal resilience category at pretest, 4 moved to the low group, while 5 moved to the high group; and out of 26 participants in the low resilience category at pretest, 12 moved to the normal group. Thus, among the 76 participants, 22% (n=17) reported an increase in perceived resilience, 11% (n=8) a decrease, while the remaining 67% (n=51) reported no change. However, the Wilcoxon signed-rank test indicated that this movement of participants across categories was statistically not significant (*P*=.11). These results are in line with those from the pretest-posttest and suggest no change in resilience among the participants.

Bivariate results for the relationship between pre-and-post difference in PSS and various demographic characteristics suggest that none of the demographic characteristics had a statistically significant association with pre-and-post difference in PSS scores (all *P*>.05; Table 4, panel a). A similar pattern of nonsignificance was observed for the pre-and-post difference in BRS scores (Table 4, panel b). The general pattern of significance observed from the bivariate analysis of PSS pre-and-post difference scores did not change when all demographic variables were simultaneously included in a multiple regression model with the PSS difference score as the outcome (all *P*>.05; Table 5, panel a). The results suggest that, while holding all else constant, the change in perceived stress between pretest and posttest did not differ significantly between (1) men and women (*P*=.15), (2) White and other race categories (*P*=.10), (3) participants identifying as Hispanic or Latino versus others (*P*=.48), (4) nurses versus other health professionals (*P*=.98), and (5) participants associated with hospitals versus those associated with other health settings (*P*=.58). In addition, there was no statistically significant association between the change in PSS score and participant age (*P*=.73). The multiple regression analysis was repeated for BRS, with the significance pattern being similar to that observed for PSS (Table 5, panel b), indicating that the pre-and-post difference in BRS scores was not significantly associated with participant gender, race, ethnicity, role, setting, or age (all *P*>.05).

**Table 4.** Bivariate results for association between scales and demographic characteristics (N=76).^a^

Demographic factor	Participants, n	Mean (SD)	Test	*P* value
(a) PSS^b^: pre-and-post difference				
Gender			Mann-Whitney *U*	.33
Female	66	1.68 (4.72)		
Male	10	0.50 (3.10)		
Race			Mann-Whitney *U*	.25
White	58	1.14 (4.51)		
Other	18	2.78 (4.54)		
Hispanic or Latino origin			Mann-Whitney *U*	.45
No	70	1.36 (4.33)		
Other	6	3.50 (6.69)		
Role			Independent samples *t*	.82
Nurse	37	1.65 (4.50)		
All else	39	1.41 (4.63)		
Setting			Independent samples *t*	.67
Hospital, urban or suburban or rural	30	1.80 (4.74)		
All else	46	1.35 (4.44)		
Age (numeric)	76	—^c^	Pearson *r*	.81
(b) BRS^d^: pre-and-post difference				
Gender			Mann-Whitney *U*	.63
Female	66	−0.13 (.61)		
Male	10	−0.05 (0.49)		
Race			Mann-Whitney *U*	.89
White	58	−0.13 (0.59)		
Other	18	−0.09 (0.61)		
Hispanic or Latino origin			Mann-Whitney *U*	.85
No	70	−0.11 (0.55)		
Other	6	−0.22 (1.02)		
Role			Independent samples *t*	.94
Nurse	37	−0.12 (0.60)		
All else	39	−0.13 (0.59)		
Setting			Independent samples *t*	.95
Hospital, urban or suburban or rural	30	−0.12 (0.44)		
All else	46	−0.13 (0.68)		
Age (numeric)	76	—	Pearson *r*	.18

a Difference measured as pretest score minus posttest score.

b PSS: Perceived Stress Scale.

c Not applicable.

d BRS: Brief Resilience Scale.

**Table 5.** Multiple regression results predicting change in scale scores (N=76).^a^

Predictors	*b* ^b^	SE(*b*)	*t* (*df*)	*P* value
(a) PSS^c^: pre-and-post difference				
Intercept	0.80	2.56	0.31 (69)	.76
Gender	−2.56	1.76	−1.45 (69)	.15
Race	2.37	1.44	1.65 (69)	.10
Hispanic	1.51	2.12	0.71 (69)	.48
Role	−0.04	1.12	−0.03 (69)	.98
Setting	−0.64	1.15	−0.56 (69)	.58
Age	0.02	0.05	0.34 (69)	.73
(b) BRS^d^: pre-and-post difference				
Intercept	−0.55	0.34	−1.62 (69)	.11
Gender	0.14	0.23	0.58 (69)	.57
Race	0.06	0.19	0.29 (69)	.77
Hispanic	−0.11	0.28	−0.38 (69)	.70
Role	0.02	0.15	0.14 (69)	.89
Setting	−0.06	0.15	−0.37 (69)	.71
Age	0.01	0.01	1.41 (69)	.16

a All independent variables are dummy-coded except age, which is numeric. Reference categories are female, White race, not Hispanic or Latino, nurse role, and hospital setting.

b *b*: regression coefficient.

c PSS: Perceived Stress Scale.

d BRS: Brief Resilience Scale.

## Discussion

### Principal Findings

The StressPal Frontline program is a training intervention that integrates mindfulness and acceptance-based components of ACT and CBT to enhance psychological flexibility and stress resilience among clinical and nonclinical health care workers. The hypothesis for this study was that completion of the StressPal Frontline program would reduce stress and improve resilience among study participants. Participants in this study exhibited a significant reduction in perceived stress, with a moderate effect on the full PSS scale. The findings of this study align with a recent umbrella review summarizing systematic reviews of various digital mental health interventions, which found positive effects on self-reported outcome measures of stress [61], in addition to anxiety, psychological well-being, and other mental health outcomes. Previous studies evaluating digital health interventions also reported small-to-medium effects on reducing stress among participants [61-63].

This study did not find a significant difference between the pretest and posttest on the BRS scale. The finding of no significant difference between the pretest and posttest on the BRS could have occurred because of the short time frame between the intervention and measurement. Previous researchers examining outcomes of resilience interventions found that improvement in resilience may have a delayed effect; impacts of interventions aimed at enhancing resilience might not be evident within short time frames and may produce significant improvement after longer periods, such as at the 4 to 5-month follow-up [64-66].

Across studies, resilience-building training methods vary considerably and may play a role in the rate of resilience-building skill acquisition. The finding at program completion of a statistically significant difference, at the item level of item analysis, in participants’ ability to “bounce back quickly after hard times” and “come through difficult times with little trouble” suggests that the learning methodologies used may have contributed to the more rapid acquisition of these resilience-strengthening skills.

Future studies should compare learning methodologies, use longitudinal designs to follow participants over time, and incorporate multiple waves of data collection to capture changes, temporal relationships, and causal pathways. Future research on digital resilience interventions should also use a randomized controlled trial design that incorporates a control group and additional outcome measures. The following psychometrically validated measures should be considered as options for outcome studies that assess psychological flexibility, cognitive defusion, mindfulness processes, stress resilience, and work engagement: Five Facets of Mindfulness Questionnaire [23,67], Work-Related Acceptance and Action Questionnaire [68], Connor-Davidson Resilience Scale [69], and the Drexel Defusion Scale [70].

### Conclusions

The StressPal Frontline program, a digital resilience health intervention that integrates mindfulness and acceptance-based components of ACT, CBT, and MBCT, was shown to have a moderate effect on reducing stress among participating clinical and nonclinical health care workers. The results of this study provide statistically significant but clinically modest evidence that digital resilience health programs focused on building psychological flexibility and resilience could be promising tools for health care delivery organizations seeking to enhance workforce well-being. The StressPal Frontline program, and similar evidence-based digital resilience health programs, could be offered to employees as part of their professional growth benefits or through employee assistance programs as one of many approaches to support the health, safety, and well-being of health care workers. Future research should evaluate the long-term effectiveness of digital resilience programs for addressing stress, resilience, and other health concerns among health care workers.

### Limitations

There are several limitations that should be considered when reviewing the results of our study. First, the pretest-posttest quasi-experimental study design did not include a separate control group, which limits our ability to rule out other explanations, such as confounding or maturation effects. Similarly, the study design may have introduced bias resulting from testing effects, which occur when participants improve from being tested twice, and/or measurement error that could occur if there were inaccuracies in measurement, such as when a participant misunderstands a question. The pretest-posttest design allowed our research team to observe and quantify changes or effects that occurred as a result of the StressPal Frontline program, which improved our ability to detect within-participant differences and reduced variability. While the pretest-posttest design has many strengths that should be noted, future research should use a randomized controlled trial as well as techniques that involve covariate adjustment.

This study also acknowledges that just because a mean difference is statistically significant, it does not mean that it is also practically significant. For this reason, it is important to interpret measures of effect size. In this study, the observed effect sizes were moderate (ie, in the small-to-medium range). While acknowledging the moderate clinical significance of the statistical results, it should be noted that from the program perspective even small reductions in perceived stress (as was the case in this study) may still be meaningful when aggregated across participants.

## Abbreviations

ACT: acceptance and commitment therapy
BRS: Brief Resilience Scale
CBT: cognitive behavioral therapy
MBCT: mindfulness-based cognitive therapy
MBSR: mindfulness-based stress reduction
PSS: Perceived Stress Scale
